# Prevalence and Risk Factors of Healthcare-Associated Infections among Hospitalized Pediatric Patients: Point Prevalence Survey in Thailand 2021

**DOI:** 10.3390/children11060738

**Published:** 2024-06-17

**Authors:** Visal Moolasart, Chaisiri Srijareonvijit, Lantharita Charoenpong, Winnada Kongdejsakda, Suvaporn Anugulruengkitt, Anond Kulthanmanusorn, Varaporn Thienthong, Sang Usayaporn, Wanwisa Kaewkhankhaeng, Oranat Rueangna, Jiratchaya Sophonphan, Weerawat Manosuthi, Viroj Tangcharoensathien

**Affiliations:** 1 Bamrasnaradura Infectious Diseases Institute, Department of Disease Control, Ministry of Public Health, Nonthaburi 11000, Thailand; chaisiri.s@bidi.mail.go.th (C.S.); lantharita.c@bidi.mail.go.th (L.C.); winnada.k@bidi.mail.go.th (W.K.); weerawat.m@bidi.mail.go.th (W.M.); 2 Division of Pediatric Infectious Diseases, Department of Pediatrics, Faculty of Medicine, Chulalongkorn University, Bangkok 10330, Thailand; suvaporn.a@chula.ac.th; 3 Center of Excellence for Pediatric Infectious Diseases and Vaccines, Chulalongkorn University, Bangkok 10330, Thailand; 4 International Health Policy Program, Ministry of Public Health, Nonthaburi 11000, Thailand; anond@ihpp.thaigov.net (A.K.); wanwisa@ihpp.thaigov.net (W.K.); oranat@ihpp.thaigov.net (O.R.); viroj@ihpp.thaigov.net (V.T.); 5 Division of International Disease Control Ports and Quarantine, Department of Disease Control, Ministry of Public Health, Nonthaburi 11000, Thailand; varaporn.thientong2@gmail.com; 6 Department of Pharmacy Practice, Faculty of Pharmaceutical Sciences, Chulalongkorn University, Bangkok 10330, Thailand; sang.u@pharm.chula.ac.th; 7 The HIV Netherlands Australia Thailand Research Collaboration (HIV-NAT), The Thai Red Cross AIDS Research Centre, Bangkok 10330, Thailand; jiratchaya.w@hivnat.org

**Keywords:** healthcare-associated infections, risk factor, point prevalence survey, and hospitalized pediatric patients

## Abstract

Background: Healthcare-associated infections (HAIs) pose a grave threat to patient safety, morbidity, and mortality, contributing to antimicrobial resistance. Thus, we estimated the point prevalence, risk factors, types, and pathogens of HAIs in hospitalized pediatric patients. Methods: A point prevalence survey (PPS) of HAIs in hospitalized pediatric patients < 18 years old was conducted from March to May 2021. Outcomes, risk factors, and types of HAIs associated with HAIs in 41 hospitals across Thailand were collected. Results: The prevalence of HAIs was 3.9% (95% CI 2.9–5.0%) (56/1443). By ages < 1 month, 1 month–2 years, 2–12 years, and 12–18 years, the prevalence of HAIs was 4.2%, 3.3%, 4.1%, and 3.0%, respectively (*p* = 0.80). Significant independent risk factors were extended hospital length of stay (LOS) and central venous catheter (CVC) use. Compared to an LOS of <4 days, LOSs of 4–7 days, 8–14 days, and >14 days had adjusted odds ratios (aORs) of 2.65 (95% CI 1.05, 6.68), 5.19 (95% CI 2.00, 13.4), and 9.03 (95% CI 3.97, 20.5), respectively. The use of a CVC had an aOR of 2.45 (95% CI 1.06–5.66). Lower respiratory tract infection (LRTI) was the most common HAI type (46.4%: 26/56). The highest prevalence of HAIs was predominantly observed in LRTI diagnoses, with the highest among these in the <1 month age category at 2.3% (17/738). Conclusion: The prevalence of HAIs in hospitalized pediatric patients was 3.9%. Extended LOS and use of CVC were HAI risk factors. A strategy for reducing LOS and reviewing insertion indications or the early planned removal of a CVC was implemented. The surveillance of HAIs stands as a cornerstone and fundamental component of IPC, offering invaluable insights that enhance hospital IPC interventions aimed at preventing HAIs.

## 1. Introduction

Healthcare-associated infections (HAIs) represent a significant menace to patient safety, contributing to increased morbidity and mortality in low- and middle-income countries (LMICs) [1,2]. Hospitalized pediatric patients face an increased risk of developing HAIs due to intrinsic factors such as their young age and the severity of the disease. Additionally, extrinsic factors like the use of invasive devices, such as central venous catheters (CVCs), endotracheal (ET) tubes for mechanical ventilation, and urinary catheters, multidrug-resistant bacteria, and the length of stay (LOS) in hospitals further contribute to this risk [3,4,5,6]. Furthermore, a prior study in China illustrated the temporal impact of age on infectious disease mortality, emphasizing its pronounced significance in the 5- to 9-year-old age group and its minimum during adolescence [7]. HAIs are key to antimicrobial resistance (AMR), and the overuse of antimicrobials promotes drug-resistant pathogens [8,9]. Protecting patients, healthcare workers, and visitors is essential for safe, high-quality care and reducing AMR risks [5,6]. Presently, numerous organizations are dedicated to enhancing the quality of surveys on HAIs or AMRs, including influential bodies like the European Centre for Disease Prevention and Control (ECDC) and the World Health Organization (WHO), both of which have protocols for the point prevalence survey (PPS) of HAIs and antimicrobial use [10,11].

As of the early 2000s, the overall prevalence of HAIs in high-income countries (HICs) ranged from 3.5 to 12%. In Europe, the average prevalence was reported at 7.1%, resulting in over four million infections annually [12], while a Canadian study of hospitalized pediatric patients demonstrated an HAI prevalence of 8.7% [13]. Reports from LMICs in Asia have varying prevalence, many of which are higher than those of HICs. Our prior research in Thailand indicated rates of 4.6% for patients less than 1 year old and 2.2% for those aged 1–17 years [14], while the reported prevalence of HAIs among hospitalized pediatric patients in India varied between 10.5% and 19.5% [15,16]. Additionally, a study in Indonesia found a higher proportion of HAIs, 17.9%, among children [17].

The surveillance of HAIs is a vital component in any comprehensive infection prevention and control (IPC) program, playing a crucial role in identifying and addressing challenging areas [18,19]. The prospective surveillance of HAIs stands as a robust standard in HAI prevention, providing timely feedback that is essential for effective monitoring [20]. Despite the substantial budget and effort involved, prospective surveillance is strategically implemented by prioritizing high-prevalence areas, such as intensive care units (neonatal intensive care unit (NICU) or pediatric intensive care unit (PICU)), or by focusing on specific infection types, such as bloodstream infections [21,22,23]. PPSs have been the preferred tool for assessing the prevalence of HAIs over recent years [24,25], regardless of the challenges posed by their cross-sectional design [26].

In Thailand, PPSs are conducted periodically every 3–4 years [14,27,28,29], with the most recent survey prior to the present study conducted 3 years ago [14]. We reported the newest survey conducted in 2021. The key focus of IPC for hospitalized pediatric patients is assessing HAI prevalence. Thus, we aimed to accomplish one primary and several secondary objectives, as follows: (1) to establish the prevalence of HAIs in hospitalized pediatric patients, as well as (2) to identify the prevalence, distribution, and associated risk factors of HAIs.

## 2. Materials and Methods

### 2.1. Study Design, Settings, and Participants

In this multicentered PPS, we surveyed HAIs among pediatric patients under 18 years old admitted to 41 healthcare facilities across Thailand. The PPS data collection occurred from March to May 2021, with data collected from each ward on a single day. Each sampled hospital contributed data within three weeks after enrollment in this study, excluding weekends or national holidays, and received ethical approval from the Ethics Committee at the Institute for the Development of Human Research Protection, Ministry of Public Health, Thailand (IHRP no. 095/2563).

This survey utilized a convenience sampling approach, as recommended by ECDC and WHO [10,11]. Forty-one hospitals with national coverage were enlisted. Thailand’s geographic regions—northern, northeastern, eastern, central, and southern—were all represented in the study design. The sampling criteria were dual, as follows: (1) ensuring each region included at least one tertiary hospital, two secondary hospitals, and two to four primary hospitals, and (2) incorporating hospitals with active infection control teams. This study included all hospitalized pediatric patients under 18 years old, utilizing systematic random sampling of admission numbers. Patients were then selected from each ward for inclusion in the survey.

### 2.2. Definitions

HAIs were defined as infections that become apparent 48 h or more after hospital admission or within 30 days following discharge following inpatient care [11]. HAI prevalence was reported as the proportion of patients with at least one HAI among hospitalized pediatric patients, as estimated using the methodology based on the Rhame–Sudderth formula [30,31]. In this study, the age groups were categorized into the following four segments: under 1 month, 1 month–2 years, 2–12 years, and 12–18 years [13]. Outcomes were stratified by hospital type (primary, secondary, and tertiary care) and hospital ownership (government, Thai Army, and private for-profit). Device-associated HAIs were defined as HAIs in patients with a relevant device in situ within 48 h from the onset of the HAIs [10]. Hospital length of stay (LOS) was defined as the date of admission to the date of the HAI in patients who had an HAI and the date of admission to the date of PPS in patients who did not have an HAI by the PPS survey date [25].

The devices considered were based on the ECDC PPS 2022–2023 [10]. The count of invasive lines included central lines, urinary catheters, tracheostomies, and ET tubes, excluding peripheral vascular catheters. In the comparison conducted by the ECDC, emphasis was placed on the correlation between an ET tube and a ventilator. The recommendation is to prioritize the monitoring of the ET tube due to a significant correlation between the ET tube and the ventilator to avoid collinearity in regression modeling [10].

### 2.3. Statistical Analysis

The survey population was analyzed descriptively as frequency and percentage with Clopper–Pearson exact confidence intervals. A chi-squared test or Fisher’s exact test was used to analyze categorical data, as appropriate. Multi-level logistic regression was used to identify risk factors for HAIs by etiologic modeling. Independent variables were level 1 data, and a random intercept was included at level 2 for hospital identity to account for different unobserved contextual effects between hospitals. The multivariate model was mostly selected from the literature and included male gender [32], age [25], and hospital level [29] as adjustment for confounding, while the potential risk factors considered included ward/unit type, LOS, CVCs, urinary catheters, ET tubes, and tracheostomy. Transferred from another hospital, surgery during admission, and admission during the last 90 days were included in the multivariate model if they were significant in univariate models due to less certainty of their potential to be risk factors. Missing data were not imputed. The statistical analysis was performed using R version 4.2.0. (R Core Team 2023, Vienna, Austria). A *p*-value of <0.05 was considered significant. We employed the Scalex and ScalaR calculators to determine sample sizes for prevalence studies. In an investigation conducted in Indonesia [17], a higher cumulative proportion of HAIs was detected in children, amounting to 17.9%. This study underscored the importance of using a minimum sample size larger than 903 when conducting surveys among pediatric hospitalized patients.

## 3. Results

### 3.1. Age and Hospital-Level Distribution of the Survey Population

**Figure 1:**
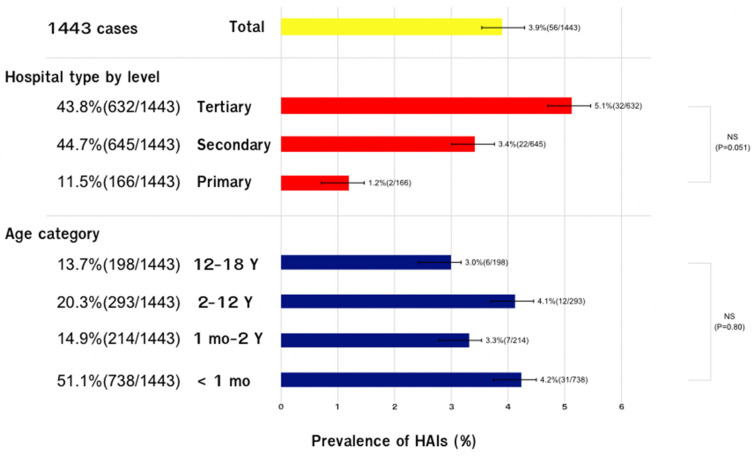
Prevalence of HAIs, proportion of hospitalized pediatric patients in 1443 hospitalized pediatric patients categorized by the proportion of hospital types (%) and age stages; age groups were defined as <1 month, 1 month to 2 years, 2 to 12 years, and 12 to 18 years. Notes: By hospital ownership type, 99.3% (1433/1443) of patients were in government-owned hospitals, 0.3% (4/1443) were in a Thai Army-owned hospital, and 0.4% (6/1443) were in a for-profit privately owned hospital. 95% CIs for the prevalence of HAIs by the following age categories are as follows: total cohort 95% CI = 2.9 to 5.0%; <1 mo age group 95% CI = 3.0 to 6.1%; 1 mo to 2 years age group 95% CI = 1.3 to 6.6%; 2–12 years age group 95% CI = 1.9 to 6.6%; and 12 to 18 years age group 95% CI = 1.1 to 6.5%. 95% CIs by hospital level were primary-level facilities 95% CI 0.1 to 4.3%; secondary-level facilities 95% CI 2.1 to 5.1%; and tertiary-level facilities 95% CI 3.5 to 7.1%. Abbreviations: NS, not significant.

The final analysis included 1443 hospitalized pediatric patients. Figure 1 shows the age distribution and number of patients by hospital level of the study population. By age distribution, 51.1% (738/1443) were less than 1 month old, 14.9% (214/1443) were 1 month to 2 years old, 20.3% (293/1443) were 2 to 12 years old, and 13.7% (198/1443) were 12 to 18 years old. By hospital level, 11.5% (166/1443) were primary-level facilities, 44.7% (645/1443) were secondary-level facilities, and 43.8% (632/1443) were tertiary-level facilities.

### 3.2. Prevalence of HAIs in the Total Cohort and Prevalence of HAIs Stratified by Age Groups

Of the total cohort of 1443 hospitalized pediatric patients, 56 children had HAIs on the survey date. The total prevalence of HAIs was 3.9% (95% CI 2.9–5.0%).

The prevalence of HAIs in children by age group was 4.2% (95% CI 3.0–6.1%) in the <1 month age group, 3.3% (95% CI 1.3–6.6%) in the 1 month to 2 years age group, 4.1% (95% CI 1.9–6.6%) in the 2 to 12 years age group, and 3.0% (95% CI 1.1–6.5%) in the 12 to 18 years age group. The prevalence of HAIs was not significantly different according to the age category (*p*-value = 0.80). The prevalence of HAIs was highest in tertiary-level care facilities at 5.1% (95% CI 3.5–7.1%), followed by secondary-level facilities at 3.4% (95% CI 2.1–5.1%), and primary-level facilities at 1.2% (95% CI 0.1–4.3%) (*p* = 0.051).

### 3.3. Prevalence of HAIs Stratified by Patient Characteristics and Exposures

Table 1 shows the characteristics of hospitalized pediatric patients and the HAI prevalence (N = 1443). The top five highest prevalences of HAIs were 16.7% (95% CI 8.6–27.9%) in the number of devices (two or more), 15.8% (95% CI 3.4–39.6%) in tracheostomy, 13.0% (95% CI 7.3–20.8%) in CVCs, 13.0% (95% CI 6.9–21.7%) in ET tubes, and 12.0% (95% CI 7.9–17.2%) in LOS (admission to HAI onset) of more than 14 days, respectively. HAIs were significantly associated with the number of devices, tracheostomy, CVC, ET tube, urine catheter, LOS, PICU/NICU, and transferred from another hospital (all *p* < 0.05). Sex, previous hospitalization within the last 90 days, and surgery since admission were not significantly associated with HAIs (all *p* > 0.05).

### 3.4. Univariate and Multivariate Analysis of Risk Factors of HAIs

Table 2 displays a univariate and multivariate analysis to identify risk factors for HAIs in the total cohort (n = 1424). Nineteen hospitalized pediatric patients were excluded for missing data on LOS. In the univariate analysis, PICU or NICU, extended LOS, CVCs, urinary catheters, tracheostomy, and ET tubes were significantly associated with HAIs (all *p* < 0.05). In the multivariate analysis, extended LOS was an independent risk factor for HAIs. The adjusted odds ratios (aORs) for extended LOS were significantly higher for patients with a hospital stay of 4–7 days (aOR 2.65, 95% CI 1.05, 6.68; *p* = 0.04), 8–14 days (aOR 5.19, 95% CI 2.00, 13.4; *p* < 0.001), and more than 14 days (aOR 9.03, 95% CI 3.97, 20.5; *p* < 0.001), compared to those with an LOS of less than 4 days. Furthermore, CVC use was identified as another independent risk factor associated with HAIs (aOR 2.45, 95% CI 1.06, 5.66; *p* = 0.04), while the urinary catheter is close to statistical significance (aOR 2.56, 95% CI 1.00, 6.59; *p* = 0.051).

Table 3 displays variations in the proportions and prevalence of HAI diagnoses across different age groups. LRTI was the most common HAI type (46.4%: 26/56). The highest prevalence of HAIs was predominantly observed in LRTI diagnoses, with the highest among these in the <1-month-old age category at 2.3% (17/738). Among the LRTI in all ages, the majority of LRTI (65.4%: 17/26) was in the <1-month-old age category.

**Figure 2:**
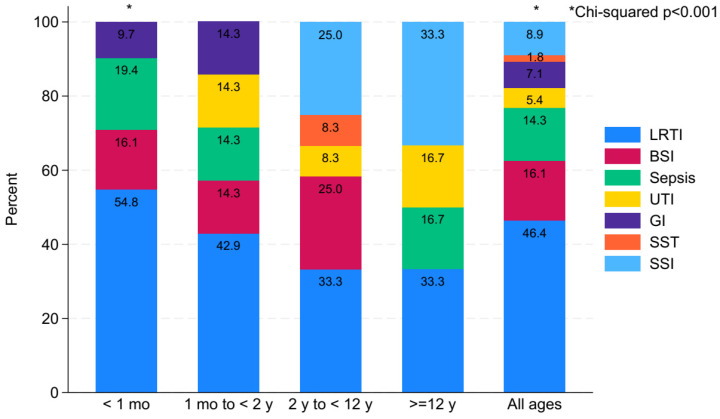
Proportions of HAI types within each age group.

Figure 2 displays the proportions of types of HAIs within each age category. In most age categories, LRTI was the most common type of HAI, with rates of 54.8% (17/31) in patients <1 month of age, with a significant difference (*p* < 0.001) of 42.9% (3/7) in those aged 1 month to 2 years and 33.3% (4/12) in those aged 2 years to 12 years. Notably, in the age group of 12 years to 18 years, surgical site infection (SSI) diagnosis (33.3%: 2/6) was equal in proportion to LRTI. In all ages, LRTI was 46.4% (26/56) of all HAIs, and there was a highly significant difference comparing the total cohort by type of infection (*p* < 0.001).

## 4. Discussion

This multicentered survey conducted a PPS for HAIs in hospitalized pediatric patients, revealing a prevalence of 3.9% across 41 randomly selected hospitals throughout Thailand. Despite the majority of hospitalized pediatric patients falling into the <1 month age group (51%), the prevalence of HAIs did not exhibit significant variations based on age categories. These newest results are similar to our team’s prior research reporting an overall HAI prevalence of 4.2%, with rates of 4.6% for patients less than 1 year old and 2.2% for those aged 1–17 years [14]. The prevalence of HAIs was higher in Canada, at 8.7%, in patients aged less than 18 years [13] and considerably higher in Indonesia, at 17.9%, in children [17]. Interestingly, a study from Indonesia, which is a neighboring country of Thailand, showed a high prevalence of HAIs. This high prevalence may be influenced by this study being conducted in a single university or referral hospital [17]. Furthermore, the prevalence of all HAIs was found to be 9.0% in Southeast Asia and 0.14% worldwide, according to systematic reviews and meta-analyses [33,34]. The prevalence of HAIs may vary due to differences in methodologies, participant inclusion criteria, and the distinct environments of the ICU, PICU, and NICU within the same hospital or across different time periods. Higher infection rates have been noted, particularly in university hospitals [35,36]. In the present study, there was a trend of increasing HAI prevalence at higher hospital levels.

The analysis revealed independent risk factors for HAIs in the present study. The prevalence of HAIs is significantly associated with an extended LOS and the use of CVC. The aORs for HAI are notably higher for patients with an LOS of 4–7 days (2.65-fold), 8–14 days (5.19-fold), and more than 14 days (9.03-fold) compared to those with an LOS of less than 4 days. Similarly, increased LOS has been associated with increased HAI risk in Indonesia, with a 5.6-fold increase in the HAI risk observed among patients with an LOS exceeding 7 days [17]. Furthermore, previous studies in children, as well as a systematic review and meta-analysis conducted in Southeast Asia, have demonstrated that HAIs are associated with an increased LOS [3,25,33,37]. A long LOS influences the high prevalence of HAIs because patients who stay longer in hospitals undergo more invasive procedures and more underlying conditions. These factors extend hospital stays and increase the risk of infection [38,39,40,41].

We identified the use of CVCs as an independent risk factor for HAIs in Thailand, with an increased risk of 2.45-fold, and urinary catheter showed a trend to significance, with an increased risk of 2.56-fold in the present study. A similar trend for CVC use was observed in an Indonesian study among children, noting a 1.8-fold increase in HAIs among CVC users [17]. This aligns with a Southeast Asian systematic review and meta-analysis identifying invasive device use as a common HAI risk factor [33]. Additionally, an earlier investigation emphasized that a substantial proportion of HAIs are linked to CVC or umbilical venous catheter (UVC) use, particularly contributing to bloodstream infections among patients < 1 month old or in pediatric intensive care units [21,22,42]. There are reasons for these elevated risks associated with the use of CVCs or UVCs. Firstly, there is extensive use of UVCs and CVCs among patients aged <1 month or in the neonatal period for many conditions, including use for administering essential drugs, fluids, parenteral nutrition, and resuscitation in the early days of life [43], whose effects on minor skin abrasions serve as entry points for pathogens, may elevate the risk of introducing pathogens into these catheters [44]. Secondly, pediatric CVC insertion poses an HAI risk, particularly in the first week. Early colonization at the insertion site, with organisms migrating along the catheter’s outer surface, is the primary cause of central line-associated blood stream infection. Beyond one week, internal colonization shifts to the direct contamination of connectors/hubs [45]. Hence, the guidelines suggest restricting the dwell time for UVC to 7–10 days. It is further advised to remove the UVC after 4 days, followed by the insertion of a peripherally inserted central catheter if a central line is still needed [46,47]. It is important to note that our study has demonstrated only a significant association of HAIs with CVCs or UVCs, while previous studies demonstrated the association of HAIs with many devices, such as ET tubes, urinary catheters, and CVCs [3,17]. This may be because the present study encompasses all hospital unit types and age categories < 18 years among hospitalized pediatric patients, while some previous studies were conducted in pediatric ICUs or high-risk areas.

The diagnosis of LRTI stands out as the most common HAI in hospitalized pediatric patients in the present study. This is concordant with a study in Turkey that demonstrated a 55% higher risk of progressing to ventilator-associated pneumonia during the neonatal period associated with a diagnosis of LRTI [48]. However, differentiating between colonization and infection poses a challenge. Previous studies indicated that 46% and 49% of tracheal aspiration samples from patients were classified as respiratory tract infections [49,50].

The strength of this study lies in its multicentered sampling and utilization of the modified ECDC and WHO protocol [10,11] for data collection under the supervision of pediatric infectious disease specialists. However, this study has limitations to address. Firstly, the validity of our careful interpretation of prevalence generated from PPS HAI prevalence should be considered because of the common issue of low sensitivity (false negatives or the underreporting of HAI) in national HAI surveillance systems [30]. Secondly, relying reliance on hospital records for data gathering raises concerns about documentation accuracy of recording, impacting this study’s findings. Data collection was carried out by local IPC experts at each site, introducing which can introduce variability across sites. Nevertheless, all data collectors underwent training and had prior experience in conducting local PPSs. Thirdly, the generalizability of the data to Thailand may be limited since only 41 hospitals were included. However, the survey covered all regions of Thailand and healthcare facilities at all levels.

## 5. Conclusions

Despite limitations, this study continues to monitor the prevalence of HAIs in pediatric patients in Thailand. It also confirms findings reported by prior studies and provides invaluable insight for strengthening hospital IPC interventions to prevent HAIs.

## Figures and Tables

**Table 1:** Characteristics of hospitalized pediatric patients and HAI prevalence (N = 1443).

Characteristic	No.	HAI Prevalence (95% CI)	*p*-Value
Patient characteristics			
Sex			0.16
Female	675	3.1 (1.9–4.7)	
Male	768	4.6 (3.2–6.3)	
Transferred from another hospital			0.003 *
No	1229	3.3 (2.3–4.4)	
Yes	214	7.5 (4.3–11.9)	
Previous hospitalization with the last 90 days			0.10
No	1322	3.6 (2.7–4.8)	
Yes	121	6.6 (2.9–12.6)	
Exposures			
Surgery during admission			0.44
No	1296	4.0 (3.0–5.2)	
Yes	147	2.7 (0.7–6.8)	
Central vascular catheter			<0.001 *
No	1335	3.1 (2.3–4.2)	
Yes	108	13.0 (7.3–20.8) ^‡^	
Urinary catheter			<0.001 *
No	1344	3.4 (2.5–4.5)	
Yes	99	11.1 (5.7–19.0)	
Tracheostomy			0.007 *
No	1424	3.7 (2.8–4.8)	
Yes	19	15.8 (3.4–39.6) ^‡^	
Endotracheal tube			<0.001 *
No	1351	3.3 (2.4–4.4)	
Yes	92	13.0 (6.9–21.7) ^‡^	
Number of devices			<0.001 *
0	1210	2.3 (1.5–3.3)	
1	167	10.2 (6.0–15.8)	
2 or more	66	16.7 (8.6–27.9) ^‡^	
Length of hospital stay			<0.001 *
<4	825	1.2 (0.6–2.2)	
4 to 7	263	3.8 (1.8–6.9)	
8 to 14	128	8.6 (4.4–14.9)	
>14	208	12.0 (7.9–17.2) ^‡^	
Missing	19	0	
PICU/NICU vs. non-PICU/NICU			<0.001 *
No	1172	2.6 (1.7–3.6)	
Yes	271	9.6 (6.4–13.7)	

Notes: ^‡^ The top five highest prevalences of HAIs. * Significant at the *p* < 0.05 level. Abbreviations: CI, confidence interval; HAIs, healthcare-associated infections; NICU, neonatal intensive care unit; PICU, pediatric intensive care unit.

**Table 2:** Univariate and multivariate hierarchical mixed effects logistic regression analysis for risk factors of HAIs in the total cohort (n = 1424).

Characteristic	cOR (95% CI)	*p*-Value	aOR (95% CI)	*p*-Value
Admission during the previous 90 days	0.55 (0.25, 1.22)	0.14		
Transferred from another hospital	2.32 (1.22–4.42)	0.01 *	1.80 (0.88, 3.69)	0.11
Ward/unit type				
Pediatric ward	Ref.		Ref.	
Surgery	1.03 (0.35–3.04)	0.96	0.99 (0.30, 3.31)	0.99
PICU or NICU	3.89 (2.16–6.99)	<0.001 *	1.41 (0.65, 3.08)	0.37
Surgery during admission	0.66 (0.23–1.88)	0.43		
Length of hospital stay				
<4 days	Ref.		Ref.	
4–7 days	3.22 (1.31–7.92)	0.01 *	2.65 (1.05, 6.68)	0.04 *
8–14 days	7.97 (3.23–19.7)	<0.001 *	5.19 (2.00, 13.4)	<0.001 *
>14 days	11.5 (5.29–25.0)	<0.001 *	9.03 (3.97, 20.5)	<0.001 *
Central vascular catheter	4.84 (2.44–9.58)	<0.001 *	2.45 (1.06, 5.66)	0.04 *
Urinary catheter	4.20 (1.88–9.39)	<0.001 *	2.56 (1.00, 6.59)	0.051
Tracheostomy	4.63 (1.23–17.5)	0.02 *	1.40 (0.31, 6.31)	0.66
Endotracheal tube	4.68 (2.29–9.57)	<0.001 *	1.48 (0.59, 3.73)	0.40

Notes: * Significant at the *p* < 0.05 level. Nineteen hospitalized pediatric patients were excluded for missing data on length of stay. The multivariate model was adjusted for male gender, age categories, and hospital level. Abbreviations: cOR, crude odds ratio; aOR, adjusted odds ratio; CI, confidence interval; NICU, neonatal intensive care unit; PICU, pediatric intensive care unit.

**Table 3:** Differences in proportions of HAI type comparing between age groups and prevalences of HAI type within age groups.

Infection Type	Age Group									
	<1 Month (n = 738)		1 Month to <2 Years (n = 214)		2 Years to <12 Years (n = 293)		≥12 Years (n = 198)		All Age Groups (N = 1443)	
	n (%)	HAI Prev.	n	HAI Prev.	n (%)	HAI Prev.	n (%)	HAI Prev.	N (%)	HAI Prev.
LRTI	17 (65.4)	2.3	3 (11.5)	1.4	4 (15.4)	1.4	2 (7.7)	1.0	26 (100)	1.8
BSI	5 (55.6)	0.7	1 (11.1)	0.5	3 (33.3)	1.0	0 (0.0)	0	9 (100)	0.6
Sepsis	6 (75.0)	0.8	1 (12.5)	0.5	0 (0.0)	0	1 (12.5)	0.5	8 (100)	0.6
UTI	0 (0.0)	0	1 (33.3)	0.5	1 (33.3)	0.3	1 (33.3)	0.5	3 (100)	0.2
GI	3 (75.0)	0.4	1 (25.0)	0.5	0 (0.0)	0	0 (0.0)	0	4 (100)	0.3
SST	0 (0.0)	0	0 (0.0)	0	1 (100.0)	0.3	0 (0.0)	0	1 (100)	0.07
SSI ^a^	0 (0.0)	0	0 (0.0)	0	3 (60.0)	1.0	2 (40.0)	1.0	5 (100)	0.4

Notes: ^a^ Significant at the <0.05 level comparing each type of infection by age group. Only first HAIs are included (n = 56). Row percentage = (%). Column percentage = [%]. Abbreviations: prev., prevalence; LRTI, lower respiratory tract infection; BSI, bloodstream infection; UTI, urinary tract infection; GI, gastrointestinal infection; SSI, surgical site infection; SST, soft tissue infection; HAI, healthcare-associated infection.

## Data Availability

The original contributions presented in the study are included in the article, further inquiries can be directed to the corresponding author. The data are not publicly available due to ethical reasons.
